# An Optimized Method for Extracting Bacterial RNA from Mouse Skin Tissue Colonized by *Mycobacterium ulcerans*

**DOI:** 10.3389/fmicb.2017.00512

**Published:** 2017-03-24

**Authors:** Marie Robbe-Saule, Jérémie Babonneau, Odile Sismeiro, Laurent Marsollier, Estelle Marion

**Affiliations:** ^1^Center for Research in Cancerology and Immunology Nantes-Angers, Institut National de la Santé et de la Recherche Médicale, Université de Nantes, Université d’AngersAngers, France; ^2^Equipe Atip-Avenir, Center for Research in Cancerology and Immunology Nantes-Angers, Institut National de la Santé et de la Recherche Médicale, Centre Hospitalier Universitaire et Université d’AngersAngers, France; ^3^Transcriptome and Epigenome Platform, Biomics, Center for Innovation and Technological Research, Institut PasteurParis, France

**Keywords:** RNA purification, RNA-seq, *Mycobacterium ulcerans*, cross-talk, host-bacteria interaction

## Abstract

Bacterial transcriptome analyses during host colonization are essential to decipher the complexity of the relationship between the bacterium and its host. RNA sequencing (RNA-seq) is a promising approach providing valuable information about bacterial adaptation, the host response and, in some cases, mutual tolerance underlying crosstalk, as recently observed in the context of *Mycobacterium ulcerans* infection. Buruli ulcer is caused by *M. ulcerans*. This neglected disease is the third most common mycobacterial disease worldwide. Without treatment, *M. ulcerans* provokes massive skin ulcers. A healing process may be observed in 5% of Buruli ulcer patients several months after the initiation of disease. This spontaneous healing process suggests that some hosts can counteract the development of the lesions caused by *M. ulcerans*. Deciphering the mechanisms involved in this process should open up new treatment possibilities. To this end, we recently developed the first mouse model for studies of the spontaneous healing process. We have shown that the healing process is based on mutual tolerance between the bacterium and its host. In this context, RNA-seq seems to be the most appropriate method for deciphering bacterial adaptation. However, due to the low bacterial load in host tissues, the isolation of mycobacterial RNA from skin tissue for RNA-seq analysis remains challenging. We developed a method for extracting and purifying mycobacterial RNA whilst minimizing the amount of host RNA in the sample. This approach was based on the extraction of bacterial RNA by a differential lysis method. The challenge in the development of this method was the choice of a lysis system favoring the removal of host RNA without damage to the bacterial cells. We made use of the thick, resistant cell wall of *M. ulcerans* to achieve this end.

## Introduction

RNA sequencing (RNA-seq), a next-generation sequencing technique, opens up unique opportunities for deciphering interactions between microorganisms and their hosts. It provides information about the relative levels of expression of the various genes and support for proteomic results. RNA-seq can also be used to identify the regulatory networks controlled by non-coding RNA, as reported for *Mycobacterium tuberculosis* (Arnvig et al., 2011).

RNA sequencing can be used to investigate and characterize the different facets of bacterial life, including, in particular, the host/microorganism tolerance underlying crosstalk between the two species (La et al., 2008; Skvortsov and Azhikina, 2010; Westermann et al., 2012; Nalpas et al., 2013; Szafranska et al., 2014; Amorim-Vaz et al., 2015). In this context, *M. ulcerans* is a fascinating microorganism, with a complex biology due to the different facets of its life cycle. *M. ulcerans* is the causal agent of Buruli ulcer, a severe cutaneous infection (Vincent et al., 2014) and the third most frequent mycobacterial disease worldwide, after tuberculosis and leprosy (Asiedu et al., 2000). *M. ulcerans* has developed sophisticated strategies for colonizing various hosts, from aquatic organisms (aquatic plants, insects, etc.) to humans, suggesting a “parasite lifestyle” (Portaels et al., 1999, 2001; Marsollier et al., 2002, 2004, 2005, 2007a,b; Johnson et al., 2007; Merritt et al., 2010; Garchitorena et al., 2014, 2015; Marion et al., 2014a, 2016a; Zogo et al., 2015; Sanhueza et al., 2016).

*Mycobacterium ulcerans* colonizes human tissues in several phases. Following the inoculation of the dermis with *M. ulcerans*, there is an intracellular phase of infection, in which the bacterium remains within macrophages and neutrophils, allowing it to evade immune system recognition (Torrado et al., 2007). *M. ulcerans* then kills the host macrophage by producing mycolactone, a lipid toxin, initiating an extracellular stage, in which local mycolactone concentrations increase considerably, leading to massive host tissue destruction. During these two stages, mycolactone is not only cytotoxic, it also modulates the immune system, modifying cytokine production and acting on the peripheral nervous system to induce the formation of a painless lesion (George et al., 1999; Coutanceau et al., 2005; Oliveira et al., 2005; Torrado et al., 2007, 2010; Silva et al., 2009; Fraga et al., 2010, 2012; Marion et al., 2014b). These pleiotropic effects of mycolactone facilitate host colonization by this bacillus. This toxin is a distinctive feature of *M. ulcerans* and seems to play a key role in its eco-epidemiology and pathogenesis.

We recently showed that 5% of Buruli ulcer patients display spontaneous healing without treatment (Marion et al., 2016a). This clinically relevant observation demonstrates that patients can develop responses that counteract the effects of *M. ulcerans* and its toxin. Deciphering the mechanisms involved in this process will open up new therapeutic strategies.

We have developed the first dedicated mouse model for studies of the spontaneous healing process (Marion et al., 2016b). During the characterization of this model, we made an interesting discovery concerning the dynamics of viable bacterial load in healed tissues: the load of cultivable bacilli was found to be both high and stable in the long term (Marion et al., 2016b). We then demonstrated that mycolactone synthesis was inhibited in healed tissues. Surprisingly, transcriptomic studies based on RT-qPCR showed that the bacteria in these tissues were not dormant. Paradoxically, transcription levels for the principal genes involved directly in toxin synthesis were unaffected, suggesting that mycolactone synthesis was regulated upstream, as already shown *in vitro* (Deshayes et al., 2013). Our previous findings suggest that RNA-seq is the most appropriate approach for deciphering the regulation of mycolactone synthesis *in vivo*.

This approach requires the isolation of large amounts of high-quality bacterial RNA, which is challenging in studies performed *in vivo*, because host RNA is much more abundant than bacterial RNA in samples. It was therefore necessary to optimize the method for extracting RNA from tissues, so as to minimize the amount of host RNA in the sample whilst ensuring the isolation of sufficient quantities of high-quality mycobacterial RNA. We present here this optimized method, based on differential lysis for the analysis of the whole transcriptome of *M. ulcerans*.

## Materials and Methods

### Ethics Statement for Animal Experiments

All animal experiments were performed in accordance with national guidelines (articles R214–87 to R214–90 from the French “rural code”) and European guidelines (directive 2010/63/EU of the European Parliament and of the council of September 22, 2010 on the protection of animals used for scientific purposes). All protocols were approved by the ethics committee of the Pays de la Loire region, under protocol nos. CEEA 2009.14 and CEEA 2012.145, and performed at the required biosafety level. Animals were maintained under specific pathogen-free conditions in the animal house facility of Angers University Hospital, France (agreement A 49 007 002).

### Bacterial Strains and Inoculation

*Mycobacterium ulcerans* strain 01G897 was originally isolated from patients from French Guiana (De Gentile et al., 1992). Bacterial suspensions were prepared as previously described (Marsollier et al., 2007b; Marion et al., 2016b), with adjustment to 2 × 10^5^ acid-fast bacilli/ml for inoculation (50 μl) into the tail of 6-week-old females of the inbred FVB/N mouse strain (Charles River Laboratories, Saint-Germain-Nuelles, France).

### RNA Extraction and Purification

RNA was extracted from infected tail skins 30 days post-infection, by the Trizol/chloroform method (Method 1), which co-extracts host and bacterial RNA, or by the differential lysis method (Method 2), optimized and adapted from that described by (Rustad et al., 2009), for the isolation of bacterial RNA alone.

### Method 1: Total RNA Extraction

**(i) Sample preparation:** Tail skin was excised from infected mice and immediately placed in a Petri dish containing a mixture of 1 ml Trizol (Ambion) and 1 ml RLT buffer (Qiagen) supplemented with 1% β-mercaptoethanol. Skin tissues were cut into smaller pieces, transferred to round-bottomed tubes and broken up with a TissueRuptor (Qiagen).

**(ii) RNA extraction and purification:** Samples were transferred to two bead beating tubes (0.1 mm glass beads, MoBio) and were shaken with TissueLyser (Qiagen) at 4°C for 5 min at 30 Hz. The samples were immediately placed on ice and centrifuged at 10,000 × *g* for 5 min at 4°C to remove cell debris. The supernatant was transferred to a 15 ml tube containing 1 ml Trizol. 200 μl chloroform/isoamyl alcohol (24:1) was added to the tube, which was then repeatedly inverted to mix and centrifuged at 10,000 × *g* for 5 min. The aqueous phase was transferred to a clean tube containing 400 μl chloroform/isoamyl alcohol and centrifuged again, as in the previous step. The aqueous phase was transferred to a clean tube containing 1 volume of 70% ethanol, and the tube was repeatedly gently inverted to mix. Total RNA was purified with the RNeasy Midi kit (Qiagen), with DNase treatment, according to the manufacturer’s protocol, and eluted in 100 μl RNase- and DNase-free water.

**(iii) DNase treatment:** Contaminating DNA was removed by retreating the RNA with DNase, for 45 min at 37°C, with the TURBO DNA-free kit (Ambion), according to the manufacturer’s protocol.

**(iv) Eukaryotic RNA removal:** To reduce the levels of contaminating host RNA from the samples, MICROBEnrich kit (Ambion) was used according to the manufacturer’s protocol.

### Method 2: Bacterial RNA Isolation by Differential Lysis Method

**(i) Sample preparation:** Tail skin was excised from infected mice and immediately placed in a Petri dish containing 2 ml Tris-EDTA (TE) buffer (10 mM Tris-HCl, 1 mM EDTA). Skin tissues were cut into smaller pieces, transferred to round-bottomed tubes and broken up with a TissueRuptor (Qiagen). 4 ml TE buffer was added and the tissue homogenates were digested with 1 ml of a 20 mg/ml proteinase K solution (Qiagen) for 10 min at 55°C (without shaking). Another 6 ml of TE buffer was added and the samples were centrifuged at 3,200 × *g* for 15 min at 4°C. The pellet, which contained the bacterial cells, was resuspended in 300 μl Tri-reagent (Zymo Research) and 300 μl RLT buffer (Qiagen) supplemented with 1% β-mercaptoethanol.

**(ii) RNA extraction and purification:** The samples were transferred into a bead beating tube (0.1 mm glass beads, MoBio) and shaken with TissueLyser (Qiagen) at 4°C for 5 min at 30 Hz. The samples were immediately placed on ice and centrifuged at 10,000 × *g* for 5 min at 4°C to remove cell debris. The supernatant was transferred into a clean tube containing 1 volume of 100% ethanol, with which it was mixed by repeated gentle inversion. The RNA was purified and treated with DNase with the Direct-zol RNA MiniPrep kit (Zymo Research), according to the manufacturer’s protocol, and eluted in 50 μl of RNase- and DNase-free water.

**(iii) DNase treatment:** Contaminating DNA was removed by retreating the RNA with DNase for 45 min at 37°C with the TURBO DNA-free kit (Ambion), according to the manufacturer’s protocol.

### RNA Analysis

**(i) Quantification and purity analysis:** Total RNA concentration and purity (A_260nm_/A_280nm_) were assessed with 1 μl of the RNA preparation, on a NanoDrop 1000^TM^ (Thermo Scientific) spectrophotometer. A ratio greater than 1.8 is usually considered to indicate satisfactory RNA purity (Imbeaud et al., 2005).

**(ii) Quality and integrity analysis:** RNA quality and integrity were assessed with the Experion automated electrophoresis system (Bio-Rad). The total RNA sample (Method 1) was diluted 10-fold and the Experion RNA StdSens analysis chip was used (quantification of 5–500 ng/μl RNA). With enriched bacterial RNA preparations (Method 2), the RNA was diluted twofold and the Experion RNA HighSens analysis chip was used (quantification of 100–5,000 pg/μl RNA). The RNA quality indicator (RQI) method returns a number between 1 (highly degraded RNA) and 10 (intact RNA) for each RNA sample (Imbeaud et al., 2005; Schroeder et al., 2006).

### Transcriptional Analysis by RT-qPCR

RT-qPCR targeting the *M. ulcerans ppk* gene was performed to detect mycobacterial RNA transcripts in RNA samples. RT-qPCR targeting the mouse *gapdh* gene was performed to evaluate the contamination of RNA samples with host RNA. The *ppk* and *gapdh* genes were selected as housekeeping genes for the RT-qPCR analyses for *M. ulcerans* and mouse, respectively. Amplification efficiency (**Table 1**) was determined from the slope of a standard curve of cDNA serial dilutions.

**Table 1:** Primer/probe sequences.

Primer/probe	Name	Sequence (5′ to 3′)	Efficiency (%)	R^2^	Reference
MuPpk_Forward	Polyphosphate kinase, MUL_1972, [*M. ulcerans* Agy99]	CGGGAAACTACAACAGCAAGACC	96%	0.9997	This study
MuPpk_Reverse		CCACCAACAGATTGCGATAGG			
MuPpk_Probe		CCGACATTGGCGCAGACCTCACC			
GAPDH_Forward	Glyceraldehyde-3-phosphate dehydrogenase, [Mouse]	GTGGCAAAGTGGAGATTGTTG	97%	0.9999	This study
GAPDH_Reverse		TGACAAGCTTCCCATTCTCG			
GAPDH_Probe		TCCACTCACGGCAAATTCAACGGCA			

**(i) Reverse transcription:** The first-strand cDNA was synthesized in a reaction volume of 20 μl containing 2 μl of total RNA, 500 ng of random primers (Invitrogen) and the M-MLV reverse transcriptase (Invitrogen). Contaminating DNA in RNA sample was checked by performing a negative control with no reverse transcriptase (RT-) for each sample.

**(ii) Quantitative real-time PCR:** qPCR was performed in a reaction volume of 10 μl containing Absolute Blue qPCR mix (Thermo Scientific), 300 nM primers, 100 nM *Taq*man probe (**Table 1**) and 2.5 μl of diluted twofold dilution of cDNA/RT-. The sequences of the primers and probes used are provided in **Table 1**. Reactions were run on a AriaMx Thermocycler (Agilent), with the following program: 10 min at 95°C and 40 cycles of 10 s at 95°C and 1 min at 60°C. Each sample was analyzed in duplicate.

### RNA-Sequencing

**(i) Ribosomal RNA (rRNA) removal:** rRNA was depleted from the mycobacterial total RNA preparations with the RiboZero Epidemiology Illumina kit, which removes eukaryote and prokaryote rRNA in a single step.

**(ii) Preparation of RNA-seq libraries:** The RNA-seq libraries were prepared with the TruSeq Stranded Total RNA LT Sample Prep kit (Illumina). The quality of all libraries was checked with the DNA-1000 kit (Agilent) on a 2100 Bioanalyzer and quantification was performed with Quant-It assays on a Qubit 1.0 fluorometer (Invitrogen).

**(iii) RNA-seq:** Clusters were generated for the resulting libraries, with Illumina HiSeq SR Cluster Kit v4 reagents. Sequencing was performed with the Illumina HiSeq 2500 system and HiSeq SBS kit v4 reagents. Runs were carried out over 65 cycles, including seven indexing cycles, to obtain 65-bp single-end reads. Sequencing data were processed with Illumina Pipeline software (Casava version 1.9). All 65-bp reads were aligned against the complete genome and plasmid sequences of *M. ulcerans* (Agy99 strain) obtained from the Burulist database with Bowtie software, and against the mouse genome (GRCm38) obtained from the Ensembl database with STAR software. Data were normalized and analyzed in R, with the Bioconductor packages.

## Results

We recently developed a model for studies of the spontaneous healing process. Our studies in this model revealed that the host response modulated toxin synthesis, providing evidence for crosstalk between *M. ulcerans* and the host. We therefore decided to develop a method for extracting bacterial RNA from host tissue in conditions suitable for high-throughput RNA-seq, to make it possible to decipher the regulation of toxin production. We evaluated two methods for isolating *M. ulcerans* RNA from host tissue (tail) for RNA-seq analysis. In this study, we inoculated the tails of mice that were then killed for analysis 30 days post-infection. All infected mice presented clinical edemas and bacterial load was estimated at about 10^6^ CFU (8.9 × 10^5^–2.1 × 10^6^ CFU). Each of the RNA extraction methods was evaluated on three samples (taken from three mice).

### Method 1: Extraction of Total RNA from Tail Skin Infected with *M. ulcerans*

**FIGURE 1:**
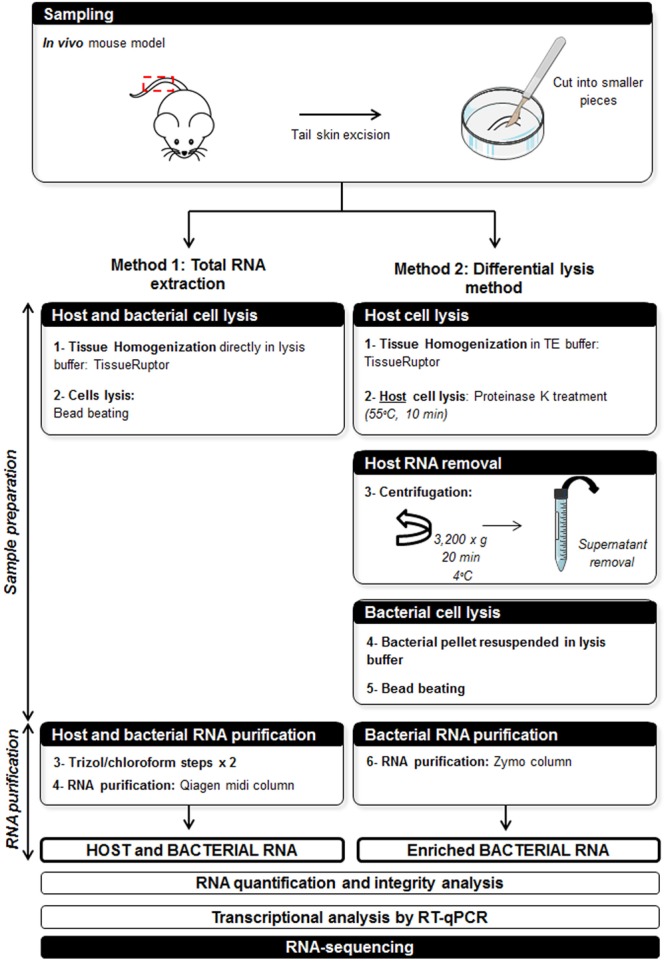
**Scheme for total RNA extraction (Method 1) and comparison with the differential lysis method (Method 2)**.

For studies of the interactions between the host and the bacteria during the infection of mice with *M. ulcerans*, we initially tried to extract bacterial and host RNA together, from the tail skin of mice infected with *M. ulcerans*. The infected tissues were broken up with a TissueRuptor and subjected to chemical and mechanical lysis, to release the bacterial and eukaryotic RNA. The RNA was then subjected to Trizol/chloroform extraction and purified on a Qiagen midi column which allows the purification of up to 1 mg RNA (**Figure 1**).

**FIGURE 2:**
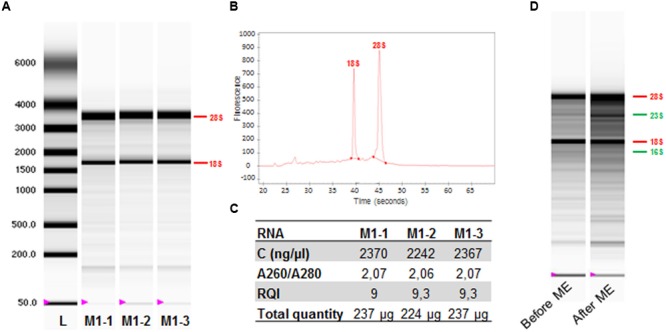
**Evaluation of the coextraction method (Method 1) (A)** Image of the electrophoresis gel for three independent RNA samples extracted from mice infected with *M. ulcerans*. L, RNA ladder. **(B)** Electropherogram: example of sample RNA derived from lane 1. **(C)** Table with RNA concentrations (ng/μl), indicator of purity (ratio A260/A280), integrity of RNA samples (RQI, RNA Quality Indicator), and total quantity (μg) of RNA extracted with Method 1. **(D)** Electropherogram: example of sample RNA before and after MicrobEnrich (ME) treatment. The 18S and 28S (mouse), and 16S and 23S (bacterial) rRNA bands are indicated in red and green, respectively.

With this approach, we obtained 200 to 300 μg of total RNA per sample (**Figure 2C**). Electrophoretic analysis revealed strong bands corresponding to the 18S and 28S rRNA of eukaryotic cells, with no bacterial RNA bands corresponding to 16S and 23S rRNA in samples (**Figure 2A** and **Supplementary Figure S1**). The electropherogram trace confirmed this result, as it contained peaks only for mouse rRNA (**Figure 2B** and **Supplementary Figure S1**). The mycobacterial RNA transcripts in samples were assessed by a reverse transcription-polymerase chain reaction (RT-qPCR) targeting the *M. ulcerans* housekeeping gene, *ppk*. The samples contained only small amounts of mycobacterial RNA, with *C*t values of 32–33 (**Table 2**). The A_260nm_/A_280nm_ ratio exceeded 1.8, confirming the purity of the RNA in each sample (**Figure 2C**), and electrophoresis showed a complete absence of RNA degradation, with an RQI value greater than 9 (**Figure 2C**). However, we were unable to assess the integrity of bacterial RNA, because no bacterial RNA was visualized.

**Table 2:** Comparison of *C*t values between the coextraction method (Method 1) and the differential lysis method (Method 2).

*C*t values	M1-1	M1-2	M1-3	M2-1	M2-2	M2-3
*M. ulcerans ppk*	33.6	32.4	32.7	30.3	30.7	31.5
Murine *gapdh*	17.7	17.5	17.1	33.3	32.5	32.8

*The values correspond to the means of duplicates.*

To eliminate the host RNA from the samples, MICROBEnrich kit (Ambion) was used which selectively removes 18S, 28S rRNA, and polyadenylated mRNA. Following MICROBEnrich treatment, electrophoresis was performed and showed this treatment to have been poorly effective in our hands, because strong bands corresponding to the 18S and 28S rRNAs were still detected (**Figure 2D**). These data confirmed the predominance of eukaryotic ribosomal RNA in samples, and suggested that mouse mRNA would probably also predominate over bacterial transcripts. However, based on our qPCR demonstrating the presence of mycobacterial RNA in samples, we decided to attempt a deep sequencing approach (RNA-seq) on a sample before and after bacterial RNA enrichment with the MICROBEnrich kit. The obtained RNA-seq data showed an absence of reads aligned with the *M. ulcerans* genome, even though mycobacterial RNA transcripts were detected by RT-qPCR, a more sensitive technique. In this case, sequencing depth was insufficient to obtain reads mapping to the *M. ulcerans* genome, confirming that the removal of mouse RNA was not effective enough to achieve an enrichment of the sample in mycobacterial RNA. In conclusion, these data demonstrate that studies of the *M. ulcerans* transcriptome *in vivo* will require a method capable of significantly decreasing eukaryotic RNA levels, to facilitate the detection of mycobacterial transcripts by RNA-seq.

### Method 2: Bacterial RNA Isolation by the Differential Lysis Method

We developed an alternative technique for the purification of bacterial RNA from infected host tissues by differential lysis, to achieve our goal. The principal challenge in this method was the choice of a lysis system resulting in host-cell lysis without damage to the bacterial cells. We recently demonstrated that the mechanical disruption of tissues had no effect on bacterial viability (Marion et al., 2016b). We therefore disrupted the host cells by mechanical lysis and treated them with proteinase K, to digest and degrade the mouse tissue whilst leaving the bacterial cells intact. The intact bacterial cells were then separated from the lysate containing host RNA by centrifugation, and the pellet containing the bacteria was resuspended in lysis buffer and subjected to bead beating. The bacterial RNA was purified on a Zymo column, making it possible to purify up to 100 μg of RNA directly from samples in Tri-reagent, a much less time-consuming approach (**Figure 1**).

**FIGURE 3:**
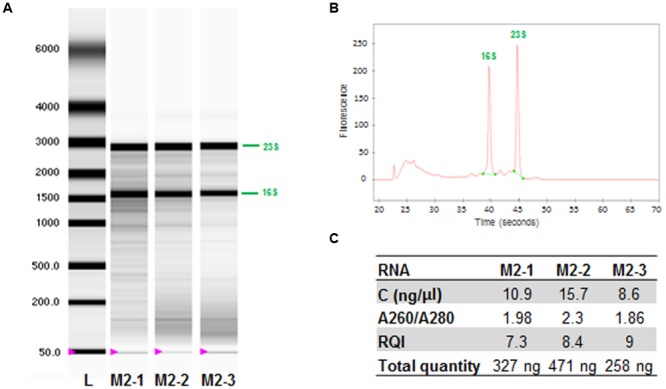
**Evaluation of the differential lysis method (Method 2). (A)** Image of the electrophoresis gel for three independent RNA samples extracted from mice infected with *M. ulcerans*. L, RNA ladder. The 16S and 23S (bacterial) rRNA bands are indicated in green. **(B)** Electropherogram: example of sample RNA derived from lane 3. **(C)** Table with RNA concentrations (ng/μl), indicator of purity (ratio A260/A280), integrity of RNA samples (RQI, RNA quality indicator), and total quantity (ng) of RNA extracted with Method 2.

We obtained 150 to 500 ng of total RNA per sample (**Figure 3C**), about one-thousandth the amount obtained with Method 1 (**Figure 2C**). However, gel electrophoresis on the total RNA obtained with this method revealed the presence of bands corresponding to the 16S and 23S bacterial rRNA whereas the mouse 28S and 18S rRNA were not detected (**Figure 3A** and **Supplementary Figure S1**). This finding was confirmed by the electropherogram trace (**Figure 3B** and **Supplementary Figure S1**).

The enrichment of samples in mycobacterial RNA by the differential lysis method (Method 2) was evaluated by RT-qPCR. The efficacy of eukaryotic RNA removal during sample preparation with Method 2 was evaluated by comparing equivalent fractions of enriched (Method 2) and non-enriched (Method 1) RNA samples. We quantified the expression of both bacterial and mouse genes in the samples obtained with the two methods.

No PCR product was obtained if the amplification was performed without prior reverse transcription (RT-). There was therefore no detectable DNA contamination. A comparison of *ppk* transcription levels showed that the transcript of this gene was present in both cases, with similar *C*t values (30–33). An analysis of murine *gapdh* gene transcription revealed a difference in *C*t of 15 between Method 1 and Method 2 (**Table 2**), corresponding to the presence of five orders of magnitude less host RNA with Method 2 than with Method 1. These results clearly demonstrate the efficacy of Method 2 for removing eukaryotic RNA. This method was, thus, considered suitable for the enrichment of samples in *M. ulcerans* RNA. The purity and integrity of bacterial RNA were assessed by determining the A_260nm_/A_280nm_ ratio and the RQI value, respectively. The A_260nm_/A_280nm_ ratio was greater than 1.8 (**Figure 3C**) and RQI values ranged from 7.3 to 9 (**Figure 3C**), indicating that the RNA was of sufficiently high quality for use in subsequent experiments, including transcriptional analyses.

**FIGURE 4:**
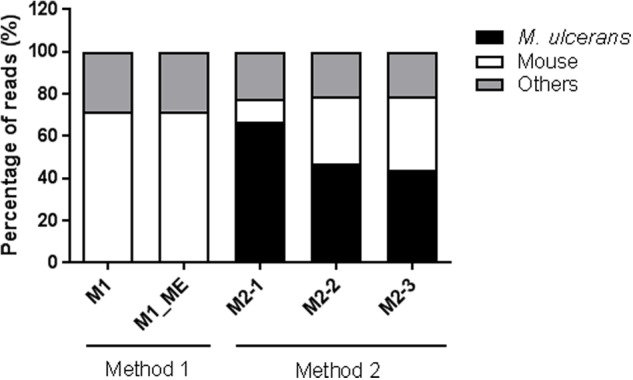
**Percentage of reads aligned with the *M. ulcerans* and mouse genomes.** M1, Total RNA sample obtained with Method 1; M1_ME, Total RNA sample obtained with Method 1 after enrichment with MICROBEnrich (ME); M2, Bacterial RNA-enriched sample obtained by differential lysis (Method 2). M2-1, M2-2, M2-3 represent three replicates for Method 2.

Given the promising nature of these findings, we performed deep sequencing (RNA-seq) on the enriched samples. The removal of rRNA is a crucial step in RNA-seq, because rRNA signals can prevent adequate coverage of the bacterial transcriptome. We therefore used the Ribo-Zero gold rRNA Removal Kit (Epidemiology, Epicentre) to remove both mouse and bacterial rRNA before producing the cDNA library. The RNA-seq data confirmed that less than 5% of the reads mapped to rRNA, demonstrating the efficacy of the rRNA removal kit. The datasets for samples M2-1, M2-2 and M2-3 obtained by the optimized method (Method 2) contained 9.5, 7.6 and 7.9 million reads aligning with the *M. ulcerans* genome, respectively, whereas no reads aligned with this genome sequence were detected with the samples obtained by Method 1 (**Figure 4**). This enrichment procedure therefore provides sufficient coverage of sequences mapping to the *M. ulcerans*, because a minimum of 2 to 5 million reads from a ribosomal RNA-depleted library is required to provide adequate coverage of the gene expression profiles of bacteria in RNA-seq experiments (Rienksma et al., 2015).

**FIGURE 5:**
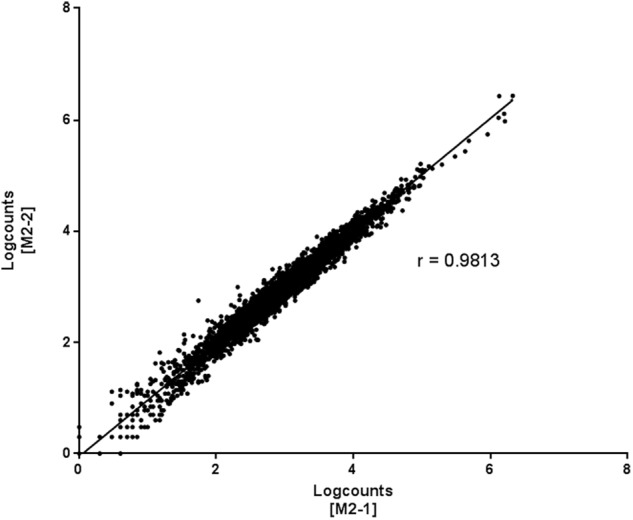
**Reproducibility of Method 2 for gene expression profiles obtained by RNA-seq.** Two biological replicates (M2-1 and M2-2) prepared with the same procedure (Method 2) were used to evaluate reproducibility in Spearman’s correlation tests. Each dot corresponds to a single gene. The *x* and *y* axes indicate the level of expression of a single gene in two different samples (M2-1 and M2-2). Spearman’s correlation coefficient = 0.9813.

Finally, two biological replicates, M2-1 and M2-2, were used to evaluate the reproducibility of the gene expression profiles obtained with RNA-seq technology. Spearman’s coefficient of correlation between samples (*r* = 0.9813) indicated a similar overall pattern of relative gene expression in the biological replicates, indicating that Method 2 was reproducible (**Figure 5**). This optimized method can therefore be used to study the mycobacterial transcriptome in a mouse model.

## Discussion

Over the last few years, RNA-seq has become a powerful tool for studies of complex interactions between microorganisms and their hosts (La et al., 2008; Skvortsov and Azhikina, 2010; Westermann et al., 2012; Szafranska et al., 2014; Amorim-Vaz et al., 2015). This method is highly suitable for studies aiming to decipher the complexity of regulation during interactions between host and bacteria.

RNA sequencing provides ready access to information about the host response during the different phases of colonization by the microorganism, but it is much more difficult to investigate transcription patterns in the microorganism. This is the principal reason for which most reports have focused essentially on host gene expression (Jenner and Young, 2005; Hossain et al., 2006; La et al., 2008). Bacterial transcriptomic signatures are much less widely documented (Jansen and Yu, 2006; La et al., 2008) because bacterial mRNA yields are much lower than those for host RNA (Lucchini et al., 2001; Hinton et al., 2004; Ferreira-Machado et al., 2015). It is therefore necessary to overcome this technical difficulty to decipher, through transcriptomic approaches, the basis of microbial attack and the ways in which the microbe escapes host defenses.

The spontaneous healing process observed during *M. ulcerans* infection highlights the ability of the host to counteract the lesion development caused by the bacterium. We recently developed the first mouse model for investigating this phenomenon (Marion et al., 2016b). Our investigations have revealed a strong inhibition of mycolactone synthesis during spontaneous healing. In conclusion, this model provides a unique opportunity to understand the regulation of mycolactone synthesis and to identify possible drug targets. To this end, RNA-seq seems to be the most appropriate method. However, due to the low bacterial load in host tissues, the isolation of mycobacterial RNA from skin tissue for RNA-seq analysis remains challenging.

In this context, we initially attempted to co-extract host and bacterial RNA together, for studies of the total transcriptome of *M. ulcerans in vivo*. This approach is hindered by a technical issue for bacterial transcriptome analysis: lower yields for bacterial RNA than for host RNA. The high abundance of host RNA reduced the coverage of sequences mapping to the *M. ulcerans* genome. Commercial kits, such as the MicrobEnrich kit, have been developed to overcome this problem, and these kits have been successfully used in some studies (Bergman et al., 2007; Audia et al., 2008; Bielecki et al., 2011; Date et al., 2014). However, in our context, use of the MicrobEnrich kit did not provide a sufficiently high level of enrichment in mycobacterial RNA, because no mycobacterial transcripts were detected by RNA-seq. This may reflect the large proportion of host RNA in sample. We therefore developed an alternative method for studying the whole transcriptome of *M. ulcerans in vivo* by RNA-seq, in which mycobacterial enrichment was increased during the sample preparation process.

This method is based on the extraction of bacterial RNA by a differential lysis method. The principal challenge in this method is the choice of a system capable of lysing the host cells without damaging the bacterial cells. Chemical or mechanical differential lysis methods are generally used for RNA isolation *in vivo* (Schnappinger et al., 2003; Talaat et al., 2004, 2007; Tuanyok et al., 2006). Our method took advantage of the thick, resistant wall of *M. ulcerans*, which is generally considered problematic for the extraction of RNA, DNA and proteins. This resistance of the bacterial wall makes it possible (i) to lyse eukaryotic cells without damaging *M. ulcerans* cells, (ii) to remove large amounts of eukaryotic RNA by simple centrifugation and, (iii) to obtain high-quality bacterial RNA for RNA-seq analysis.

## Conclusion

We have developed the first simple protocol for the selective extraction of *M. ulcerans* RNA from host tissues. This method represents a significant improvement as it provides RNA of sufficiently high quality for RNA-seq analysis. This strategy will make it possible to perform *in vivo* studies of the interactions between mycobacteria and their host, improving our understanding of the molecular mechanisms underlying infection. Finally, it may be possible to adapt this method for the isolation of other mycobacteria in the host colonization context.

## Author Contributions

MR-S, LM, and EM conceived and designed the experiments. MR-S, JB, OS, and EM performed the experiments. MR-S, OS, LM and EM performed data analysis. MR-S and LM wrote the paper.

## Conflict of Interest Statement

The authors declare that the research was conducted in the absence of any commercial or financial relationships that could be construed as a potential conflict of interest.

## Supplementary Material

FIGURE S1 **RNA profile of a mixed sample containing bacterial and mouse RNA (M+B), a mouse sample (M) and a *M. ulcerans* sample (B). (A)** Image of the electrophoresis gel for the RNA samples. **(B)** Electropherogram of RNA samples. The 18S and 28S (mouse), and 16S and 23S (bacterial) rRNA bands are indicated in red and green, respectively.
