# Supplementary figures and images for: An Optimized Method for Extracting Bacterial RNA from Mouse Skin Tissue Colonized by Mycobacterium ulcerans
Source: Front Microbiol. 2017 Mar 24;8:512. doi: 10.3389/fmicb.2017.00512 (PMC5364165; doi:10.3389/fmicb.2017.00512)

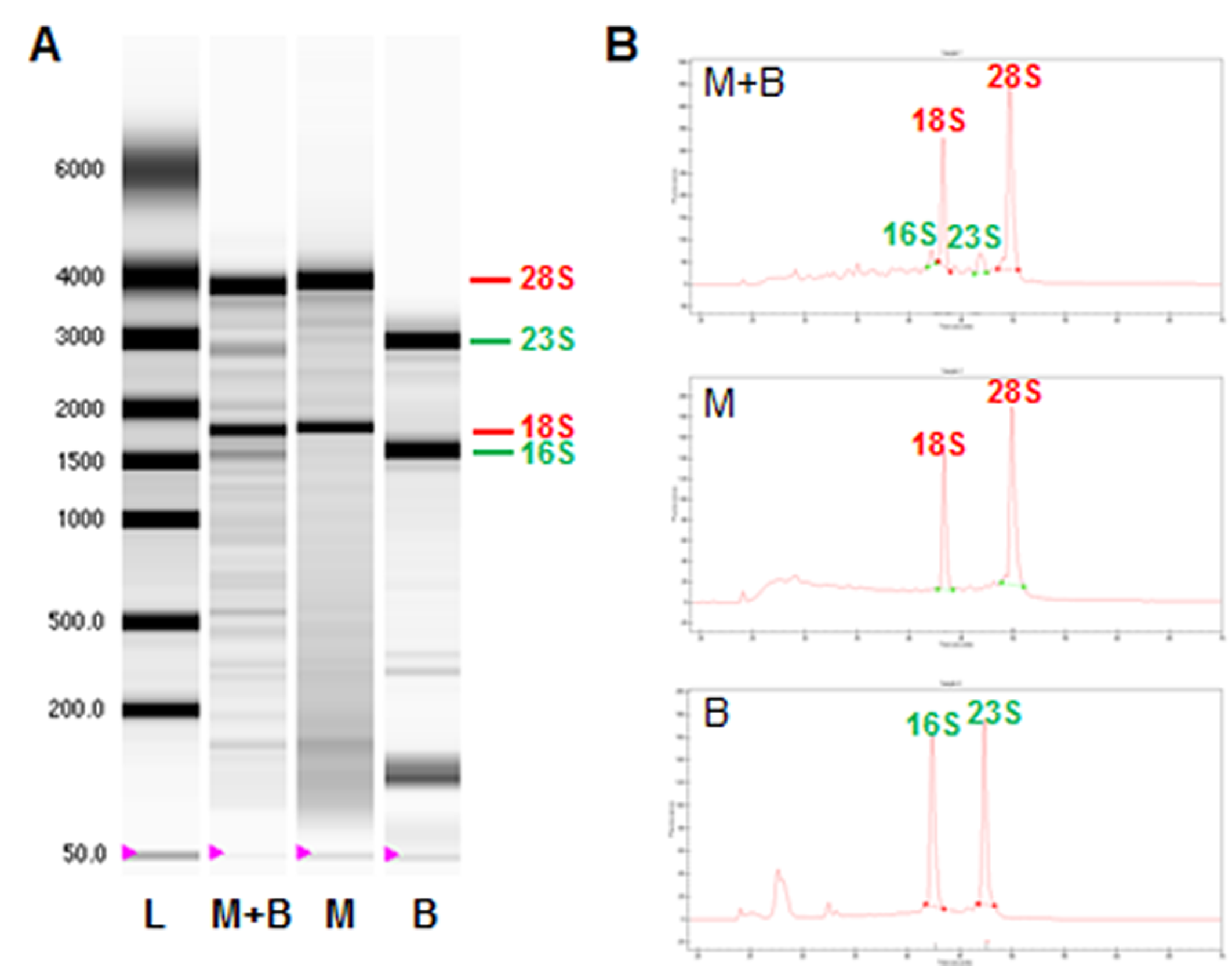

Supplement: FIGURE S1 — RNA profile of a mixed sample containing bacterial and mouse RNA (M+B), a mouse sample (M) and a M. ulcerans sample (B). (A) Image of the electrophoresis gel for the RNA samples. (B) Electropherogram of RNA samples. The 18S and 28S (mouse), and 16S and 23S (bacterial) rRNA bands are indicated in red and green, respectively. [file Image_1.TIF]
